# Orientin suppresses osteoclastogenesis and ameliorates ovariectomy‐induced osteoporosis via suppressing ROS production

**DOI:** 10.1002/fsn3.3516

**Published:** 2023-06-28

**Authors:** Yan Zheng, Xing Wang, Ya‐Jing Pan, Xiao‐Feng Shi, Lei Yang, Yong‐Liang Lou

**Affiliations:** ^1^ Wenzhou Key Laboratory of Sanitary Microbiology, Key Laboratory of Laboratory Medicine, Ministry of Education, School of Laboratory Medicine and Life Sciences Wenzhou Medical University Wenzhou China; ^2^ Department of Endocrinology Affiliated Yueqing Hospital Wenzhou China; ^3^ Department of Orthopedic The Second Affiliated Hospital and Yuying Children's Hospital of Wenzhou Medical University Wenzhou China

**Keywords:** NF‐κB/Nrf2 pathway, Orientin, osteoclast, osteoporosis, reactive oxygen species

## Abstract

The aberrant differentiation of osteoclasts is a key feature of the pathogenesis of osteoporosis, which has a devastating impact on human health. While the effects of Orientin (Ori) on osteoporosis, particularly on RANKL‐stimulated osteoclast production and activation, remain still unclear, Ori has been found to display several biological activities, including antioxidant and anti‐inflammatory. In this work, we investigated the possible pathways through which Ori suppressed RANKL‐induced osteoclast development and showed for the first time that it does so. The macrophages from the bone marrow (BMMs) were cultivated and then treated with Ori after being stimulated with RANKL. Then, TRAP‐positive multinucleated cells were counted, and F‐actin ring analysis was used to assess Ori's impact on mature osteoclast development. In addition, dihydroethidium (DHE) staining was used to evaluate the impact of Ori on RANKL‐induced reactive oxygen species (ROS). In addition, we performed western blotting and quantitative RT‐PCR analysis to investigate probable causes of these downregulation effects. We discovered that Ori inhibits the creation of osteoclasts, the gene and protein expressions unique to osteoclasts, and the ROS production. By activating Nrf2 and other ROS‐scavenging enzymes, Ori reduces intracellular ROS levels. The expression of the main transcription factor of osteoclast development, c‐Fos, was downregulated together with NFATc1, CTSK, and NFATc2, thanks to Ori's inhibition of RANKL‐induced NF‐κB. Consistent with its in vitro antiosteoclastogenic action, Ori therapy in the ovariectomized (OVX) rat model was also able to restore bone mass and improve microarchitecture in the distal femurs. Together, our results demonstrate that Ori is a flavonoid molecule with therapeutic promise for bone illnesses associated with osteoclasts, such as osteoporosis.

## Graphical abstract

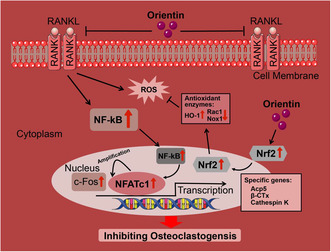

## 1 INTRODUCTION

Osteoporosis is caused by a number of reasons and is defined by an unfavorable ratio of bone mineral synthesis to resorption. These processes are mediated, respectively, by osteoblasts and osteoclasts (Vrathasha et al., 2019; Yang et al., 2019). Multinucleated large cells called osteoclasts have their origins in the monocyte/macrophage lineage (Xiong et al., 2016). Their improper differentiation or activity may result in a number of skeletal disorders, including Paget's disease of bone, periprosthetic osteolysis, bone tumors, osteoporosis, and osteopetrosis (Marcoline et al., 2016). As a result, blocking the development and activity of osteoclasts is seen as a promising approach for dealing with disorders characterized by excessive bone resorption caused by osteoclasts (Ruan et al., 2012). Bisphosphonates and denosumab are used often nowadays as part of estrogen replacement treatment to reduce osteoclast activity (Kim et al., 2019). Despite being effective therapeutically, these medications do not prevent potentially fatal side effects (Hadaya et al., 2019). Consequently, the search for novel medications to heal illnesses caused by osteoclast‐mediated bone destruction that have minimum adverse effects continues.

Bone‐destroying osteoclasts come from the multinucleated, mononucleate monocyte–macrophage hematopoietic lineage. Additionally, macrophage colony‐stimulating factor (M‐CSF) and RANKL are essential for osteoclast differentiation. Osteoclast precursor cells have been shown to respond favorably to M‐CSF, which has been shown to increase their proliferation and survival. By attaching to its receptor, RANK, RANKL plays a critical role in regulating mature osteoclast development, function, and survival. Moreover, osteoclastogenesis relies on the transcriptional activity of NFATc1, cathepsin K (CTSK), and c‐Fos, all of which are triggered by the nuclear factor‐κB (NF‐κB) signaling pathways, which are mobilized in part thanks to RANK recruitment (Yu et al., 2015; Zhao et al., 2020). Therefore, it is imperative to suppress RANKL‐induced downstream signaling pathways.

Bone metabolism also involves reactive oxygen species (Zhang, Cui, et al., 2020). Multiple studies have shown that reactive oxygen species (ROS) may also influence osteoclast production and activity, leading to increased bone resorption, decreased trabecular bone mass, and severe bone loss owing to insufficient levels of antioxidant enzymes. Serum oxidative stress biomarker levels are substantially greater, and antioxidant levels are much lower in postmenopausal women with osteoporosis, according to a recent study (Xiao et al., 2020). In addition, the risk of osteoporosis rises when the bone's defensive systems against oxidative stress are weakened after estrogen discontinuation (Cervellati et al., 2013). In response to RANKL, osteoclast precursors generate ROS through the RANK, Ras‐related C3 botulinum toxin substrate 1 (Rac1), and NADPH oxidase 1 (Nox1) cascades. Multiple investigations have shown that blocking ROS generation inhibits osteoclastogenesis (Wada et al., 2020). Reducing ROS requires the production of antioxidant enzymes like heme oxygenase‐1 (HO‐1) and Catalase, and this is exactly what the nuclear factor erythroid 2‐like 2 (Nrf2) does. Decreased intracellular ROS levels, transcription of antioxidant enzymes, and dramatically inhibited osteoclast formation were the results of increasing nuclear accumulation of Nrf2 (Zhang, Shang, et al., 2020). In this way, suppressing osteoclastogenesis relies on preventing BMMs from producing active oxygen.

Many different types of edible plants, including Luteolin, Apigenin, and Ocimum sanctum, contain the bioactive component Orientin, which is a member of the apigenin‐8‐C‐glucoside family (Thangaraj et al., 2019). Various scientists are interested in studying Ori because of its various bioactive qualities, which include antioxidation, anti‐inflammation, psychiatry protection, cardio‐protection, and anxiolytic (Lam et al., 2016; Zhang et al., 2022). Recent research has also shown various benefits, such as decreased body fat, protection against renal pyrosis, and prevention of stroke (Choi et al., 2020; Jing et al., 2020). Ori has been shown to have several beneficial effects in the fields of antioxidant and anti‐inflammatory molecular research. The attenuation of brain ischemia/reperfusion damage by Ori was reported by Wang et al. through the NF‐κB signaling route, and the attenuation of lipopolysaccharide‐induced liver injury by Li et al. via the suppression of oxidative stress via the Nrf2 pathway (Li et al., 2022; Wang et al., 2017). It is unclear, however, whether or not Ori has an inhibitory impact on RANKL‐induced osteoclastic differentiation or whether or not it improves oxidative stress in osteoclasts. Therefore, the purpose of this research was to investigate the impact of Cor and its molecular function on RANKL‐induced osteoclast survival, differentiation, and function. The role of Ori in the development of osteoclast‐related illnesses was further elucidated using an ovariectomized rat model in the area of osteoporosis models. Ori prevents bone loss caused by OVX by inhibiting NF‐κB pathways, hence decreasing RANKL‐induced osteoclastogenesis and reducing ROS formation through upregulation of ROS‐scavenging enzyme expression mediated by Nrf2. To further understand the molecular processes at play during osteoclastogenesis and to determine if Ori plays a role in this process, we conducted an in vitro investigation.

## 2 MATERIALS AND METHODS

### 2.1 Materials and reagents

Orientin (purity >98%, batch No. HY‐N0405, CAS No. 28608–75‐5) and dimethyl sulfoxide (DMSO) were acquired from MedChemExpression company (Monmouth Junction, US). Both RANKL and M‐CSF were purchased from Research and Development (Minneapolis, Minnesota, USA) and diluted as directed by the manufacturer. Gibco was used to get fetal bovine serum (FBS) and minimal essential medium (Gaithersburg, MD, USA). Sigma was used for the purchase of the Cell Counting Kit‐8 (CCK‐8) assay kit, TRAcP staining reagents, and Phalloidin‐FITC (Saint Louis, US). Cell Signaling Technology (Danvers, MA, USA) provided both the primary antibodies (NFATc1, CTSK, HO‐1, and GAPDH) and the secondary antibodies. Abcam (Cambridge, UK) supplied the primary antibodies for Nox1, Rac1, n‐Nrf2, and Lamin B; p‐p65, p65, and IκB were purchased from Santa Cruz Biotechnology (Dallas, CA, USA). No less than analytical‐ or cell‐culture‐grade reagents were employed in any of the studies.

### 2.2 Ethical treatment of animals

Rat experiments were conducted in accordance with Wenzhou Medical University's guidelines for the care of experimental animals, and they were all approved by the institution's Animal Care and Welfare Committee (SYXK2020‐0004).

### 2.3 Isolation of cells and cytotoxic assay

C57BL/6J mouse bone marrow stromal cells (BMMs) were transplanted into α‐MEM‐coated culture flasks containing 100 U/mL penicillin and 100 μg/mL streptomycin, FBS (10%), and M‐CSF (50 ng/mL). One to three generationally passed cells were employed in the assays that followed. Ninety‐six‐well plates containing 5 × 10^3^ BMMs each were planted with the cells to test for cytotoxicity. Cells were exposed to the specified Ori dosages (0, 5, 10, 20, 40, 80, 160, and 320 μM) after 24/48 h after adhering to them over the previous night. Incubation of the cells in the CCK‐8 reagent for 2 h was performed. At 450 nm, a spectrophotometer (Thermo Fisher Science, Waltham, MA, USA) was chosen to measure all of the samples.

### 2.4 In vitro test for osteoclast differentiation

At a density of 5 × 10^3^ cells/well, BMMs were grown on 96‐well plates overnight. To induce osteoclast differentiation, the cells were cultured in a complete MEM medium supplemented every other day with either 40 μM Ori or vehicle control (DMSO). The cells were cultured for 5 days before being fixed in 4% paraformaldehyde (PFA) and stained for TRAcP using a TRAcP staining kit (Sigma, Saint Louis, USA) as per the manufacturer's instructions (Jansen et al., 2020). In order to determine the number of osteoclasts, TRAcP‐positive cells with more than three nuclei were counted.

### 2.5 Staining for F‐actin ring

To determine if Ori had an impact on the creation of mature F‐actin rings and to see how sealing zones arise in BMMs, we stained the cytoskeletal fibrous actin (F‐actin). The procedure included washing the cells with PBS, fixing them in 4% paraformaldehyde for 15 min, washing the cells three more times, using Triton X‐100 (0.1%) for 15 min to permeabilize the cells, and staining them with Rhodamine‐phalloidin in the dark for 1 h. Cells were treated with DAPI for 10 min to stain the nuclei. For image analysis, cytoskeletons stained with F‐actin were seen and measured using a Nikon fluorescent microscope.

### 2.6 Assay for bone resorption

After 3 days of RANKL stimulation, bovine bone slices were implanted with equal numbers of BMM‐derived preosteoclasts and treated with Ori (40 μM) for an additional 48 h. Then, sonication and mechanical agitation were used to dislodge cells that had stuck to the bone slices. Using ImageJ software, the bone resorption area was measured after resorption pits were seen using an SEM (FEI Quanta 250).

### 2.7 qRT‐PCR

To extract RNA, cells from various treatment groups were lysed using RNAiso Plus (Takara Bio, Otsu, Japan) following the manufacturer's requirements after being rinsed with cold PBS. After then, cDNA was produced by reverse‐transcription of the whole RNA. Using an RT‐PCR Detection System (CFX96, Bio‐Rad, Hercules, CA, USA), quantitative real‐time PCR was carried out using the cDNA. The values were adjusted to reflect GAPDH levels. The genes' primer sequences are in appendix.

### 2.8 Western blotting

Different sets of cultured cells were given three 5‐min cold PBS washes between treatments. The cells were lysed for 20 min at 4°C in RIPA lysis buffer (Biyuntian, Hangzhou, China). The collected cell lysates were then centrifuged (10,000 × g, 10 min). Loading buffer was used to dissolve the acquired supernatants. Proteins from a 10‐μL sample were isolated on 10% SDS‐PAGE gels and transferred to PVDF membranes. (Bio‐Rad, Hercules, CA, USA). After blocking the membranes at room temperature for 2 h with 5% nonfat dry milk, they were washed twice for 10 min in TBST20. Primary antibodies were applied to the membranes and incubated at 4°C for 24 h, followed by incubation with secondary antibodies at room temperature for 2 h. An electrochemical luminescence reagent (Millipore, Billerica, MA, USA) was chosen to detect the protein bands. ImageJ was used to count the number of grays throughout each band.

### 2.9 Detection of ROS production

DHE staining and MitoSox were performed to measure intracellular ROS and mitochondrial ROS, respectively. There was a 15‐min fixation in 4% formaldehyde followed by a PBS wash of the cells. The cells were incubated for 30 min at 37°C after the addition of DHE and MitoSox. After two rounds of PBS washing, fluorescence pictures were captured using a Nikon microscope. The level of fluorescence was measured.

### 2.10 Docking analysis

Protein database (https://www.rcsb.org/structure) provides crystal structure of the protein of interest (Nrf2 and p‐p65). The chemical structure of Orientin was obtained from the PubChem database (https://pubchem.ncbi.nlm.nih.gov/).

The protein target serves as a receptor, while the modified chemical is employed as a small molecule ligand. The Grid Box's origin, breadth, depth, and height are all established by the nature of the tiny molecule's interaction with its target. In the end, AutoDock was used for batch docking, and the results of molecular docking were examined. PyMOL 2.1 was used to create images of the compound's and protein's binding effects. A Lamarckian genetic method for molecular docking is applied in the computation procedure. The parameters of the method are as follows: 150 population, maximum of 25 million energy assessments, maximum of the 2000 s, 0.8 crossover rate, 0.02 mutation rate, 50 separate docking runs, and evaluation of the docked structure based on the combined free energy.

Once molecular docking is complete, the top‐ranked compounds are visualized using PyMOL, and their manner of contact with the target protein and active site residues (including hydrogen bond interaction, π–π interaction, and hydrophobic interaction) are analyzed.

### 2.11 Ovariectomized rat model

There are separate Ethics Committees for patients at Wenzhou Medical University's Yuying Children's Hospital and the Second Affiliated Hospital, both of which have given their blessing to the procedure for animal research. Fifteen female SD rats, aged 3 months and weighing 250 ± 20 g, were obtained from the Animal Center of the Chinese Academy of Science in Shanghai, China. The rats were divided into three groups, with five rats in each group: sham‐operated rats, OVX rats, and OVX rats treated with Ori (40 mg/kg). After 1 week of adaptive feeding, surgical removal of the ovaries was performed on both OVX rats and Ori‐treated OVX rats using a 10% chloral hydrate solution, whereas just one ovary was grown in the sham‐operated rat. PBS or Ori (40 mg/kg) was administered intraperitoneally every other day for 8 weeks, beginning 1 week after surgery. One day following their last dose, all of the rats in the study were put to sleep. Following their removal, the femurs were subjected to a 24‐h preservation period in 10% formalin. Subsequently, they underwent radiological examination through the use of micro‐computed tomography (micro‐CT) and histomorphometric bone analysis.

### 2.12 Scanning for micro‐CT

Using a high‐resolution micro‐CT scanner, three‐dimensional pictures of the distal femur were created (Skyscan 1176; Skyscan; Aartselaar, Belgium). Using software in the micro‐CT workstation and three‐dimensional reconstructed pictures, the volume of interest's (VOI) microarchitectural characteristics were determined. Finally, quantitative measurements of characteristics relevant to the area of interest (ROI), such as the trabecular number (Tb. N, 1/mm), trabecular thickness (Tb. Th, mm), bone volume to tissue volume (BV/TV), and trabecular separation (Tb. Sp, mm), were made to evaluate the quality of the bone.

### 2.13 Labeling for calcein double

Animals were doubly labeled before sacrifice by receiving 30 mg/kg of calcein. We performed the previously mentioned measurement of the mineralization apposition rate (MAR) (Jin et al., 2023).

### 2.14 Histomorphology

Following CT scanning, for decalcification of the distal femur, 10% EDTA was used and replaced once a week for 8 weeks. This was followed by paraffin wax embedding, ethanol fixation, xylene clearance, and another paraffin wax embedding. Sections of the bones that were 4 μm thick and longitudinally sliced were then put on polylysine‐coated glass slides and stained with Masson's trichrome, H&E, and TRAcP.

### 2.15 Serum ELISA assay

To obtain the serum, blood samples from each rat were centrifuged at 1000 × g for 10 min at room temperature. Using the appropriate ELISA kit (Elabscience, Wuhan, China), the levels of the bone resorption biomarkers TRAcP5 and β‐CTx were determined.

### 2.16 Analytical statistics

Every piece of information offered comes from at least three different experiments. The data are presented as means ± standard error of the mean (SEM). Statistical significance was evaluated using Student's *t*‐test and ANOVA. One‐way ANOVA was applied for comparisons between more than two groups, while the Student's *t*‐test was used to assess differences between the two groups. Furthermore, two‐way ANOVA was employed to analyze the effects of various treatments. It was considered statistically significant if *p* < .05.

## 3 RESULTS

### 3.1 Ori reduces osteoclast differentiation and blocks RANKL's effects on the creation of F‐Actin belts and osteoclasts' ability to erode bone

**FIGURE 1:**
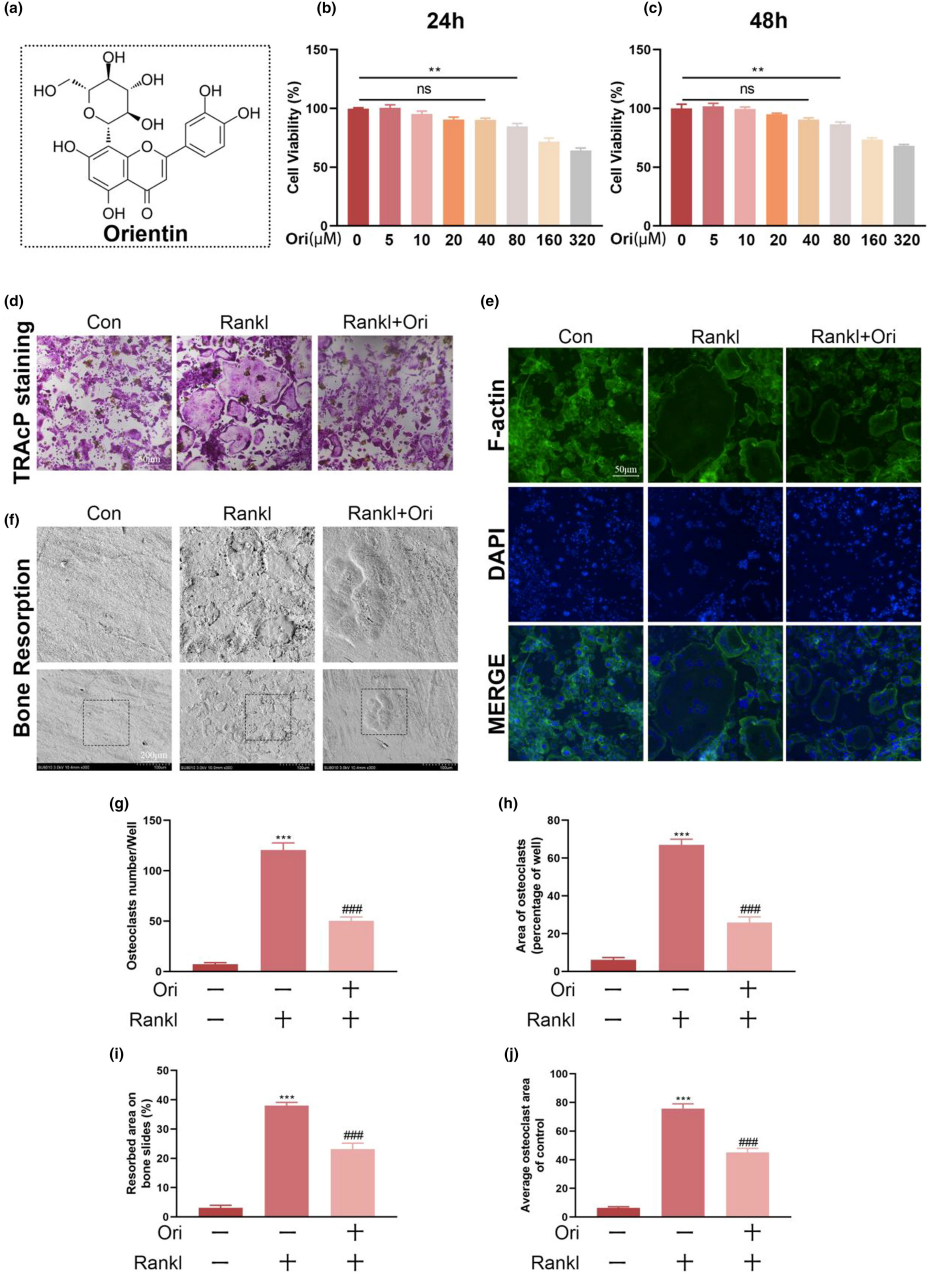
Orientin inhibits the osteoclastogenesis and resorption functions induced by RANKL in vitro. (a) Orientin's chemical structure. (b and c) At 24 h and 48 h, Orientin's cytotoxic effect on BMMs was evaluated using the CCK‐8 cytotoxicity assay. (d, g, and h) Representative images and quantitative analysis of TRAcP staining in BMMs treated differentially. (e and j) Fluorescence microscopy was used to acquire images of F‐actin (green) and nuclei (blue), as well as quantitative analysis of BMMs that were treated differently. (f and i) Quantitative analysis of scanning electron micrographs (SEM) of bone sections seeded with osteoclasts (scale bar, 100 μm). There were at least three replicates of each experiment. The numbers in the figures are the means ± SEM of three independent measurements. Statistical significance between the test and control groups: **p < .01, ***p < .001. When compared to the RANKL groups, ^##^ *p* < .001 (ns = not significant).

To determine the impact of Ori on cell viability (Figure 1a), various Ori concentrations were applied to BMMs for 24 or 48 h. As can be seen in Figure 1b,c, treating cells with Ori at concentrations below 80 μM had no effect on the viability of the cells. So, in all following vitro tests, 40 μM of Ori was employed. Next, we looked at whether Ori inhibited the production of F‐actin. For bone resorption, F‐actin forms bone‐surface loop structures. Phalloidin‐Alexa Fluor 488 labeling of osteoclasts without Ori treatment exhibited distinctive F‐actin rings, as illustrated in Figure 1e,f. On the other hand, after receiving the Ori therapy, the F‐actin ring regions decreased, and more pleomorphic F‐actin rings emerged. In addition, the primary role of osteoclasts is bone resorption, which is also a significant contributor to bone loss. So, we further investigated if Ori prevents osteoclasts from resorbing bone. Figure 1g demonstrates that treatment with Ori (40 μM) decreased the region of resorption as compared to the RANKL treatment group (Figure 1h). These findings demonstrate that Ori suppresses the production of F‐actin, osteoclastogenesis, and bone resorption.

### 3.2 Ori inhibits the expression of osteoclast‐related genes and proteins

**FIGURE 2:**
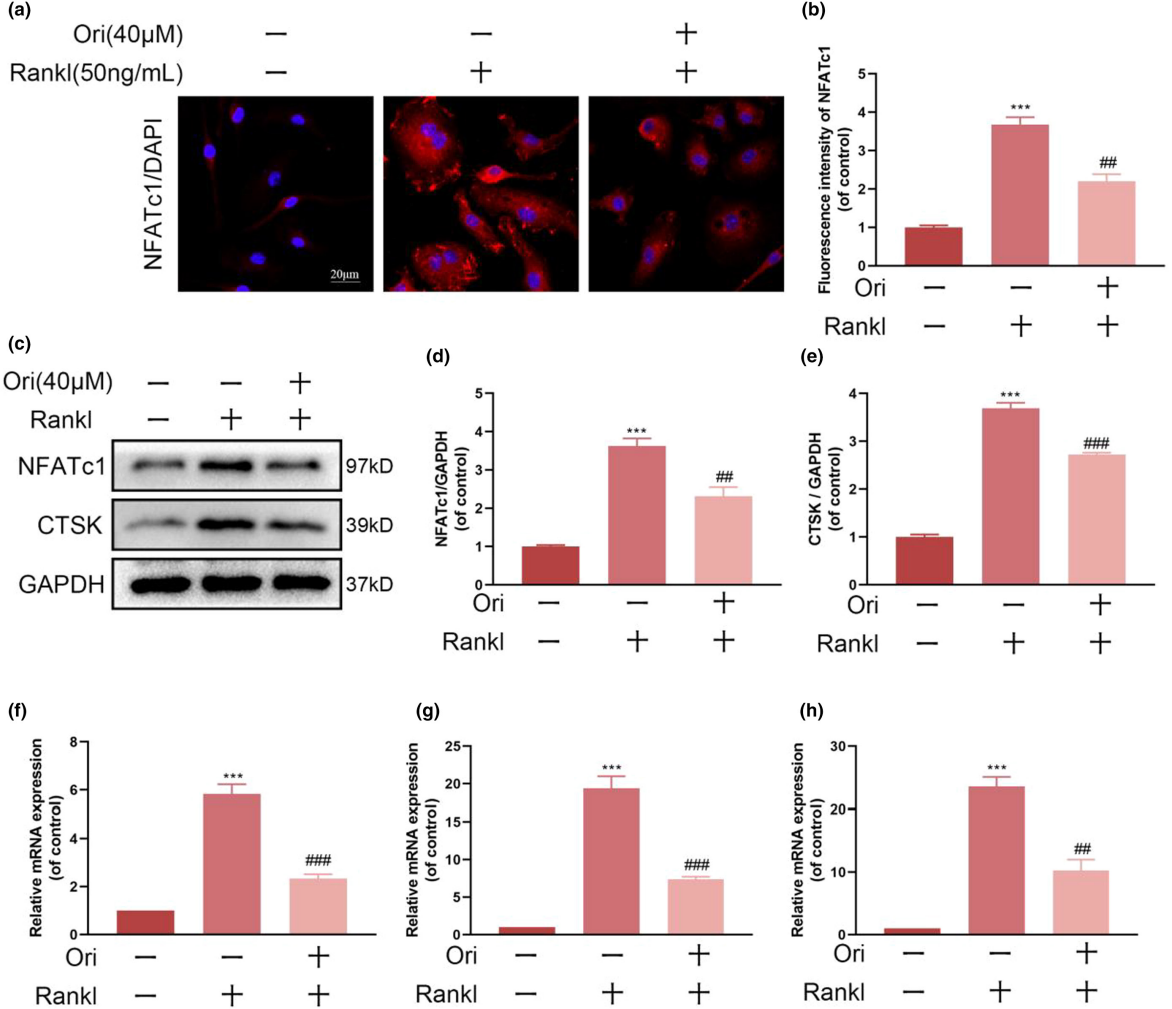
Orientin inhibits RANKL‐induced osteoclastogenesis‐related proteins and gene expression. (a and b) Images of differently treated BMMs and quantitative measurement of their NFATc1 fluorescence. (c–e) Western blotting was used to examine the BMMs that received various treatments for the amounts of NFATc1 and CTSK protein expression. (f–h) Gene expression profiles of BMMs exposed to various treatments for NFATc1, CTSK, and c‐Fos. Averages ± SEM from three replicates are shown in the figures. Statistical significance between the test and control groups: ***p < .001. When compared to the RANKL groups, ^##^ *p* < .01, ^###^ *p* < .001.

To gain a deeper understanding of Ori's impact on osteoclast development and function, we utilized western blotting and quantitative polymerase chain reaction techniques. Specifically, we analyzed the expression levels of osteoclast‐specific genes such as NFATc1, c‐Fos, and CTSK at the protein and mRNA levels. Ori‐treated groups showed significant inhibition of RANKL‐induced osteoclast‐specific gene expression compared to the RANKL group (Figure 2a,b). Western blotting experiments also showed that this drug suppressed NFATc1 and c‐Fos protein expression (Figure 2c,d). The expression of genes unique to osteoclasts requires NFATc1 self‐amplification and nuclear translocation during RANKL‐induced osteoclastogenesis. Treatment with RANKL dramatically boosted NFATc1 nuclear translocation, whereas Ori greatly inhibited it (Figure 2e,f). These findings provide further proof of Ori's suppressive effects on osteoclasts.

### 3.3 Ori decreases ROS generation during osteoclastogenesis triggered by RANKL through modulation of the NF‐κB/Nrf2 pathway and other antioxidant enzymes

**FIGURE 3:**
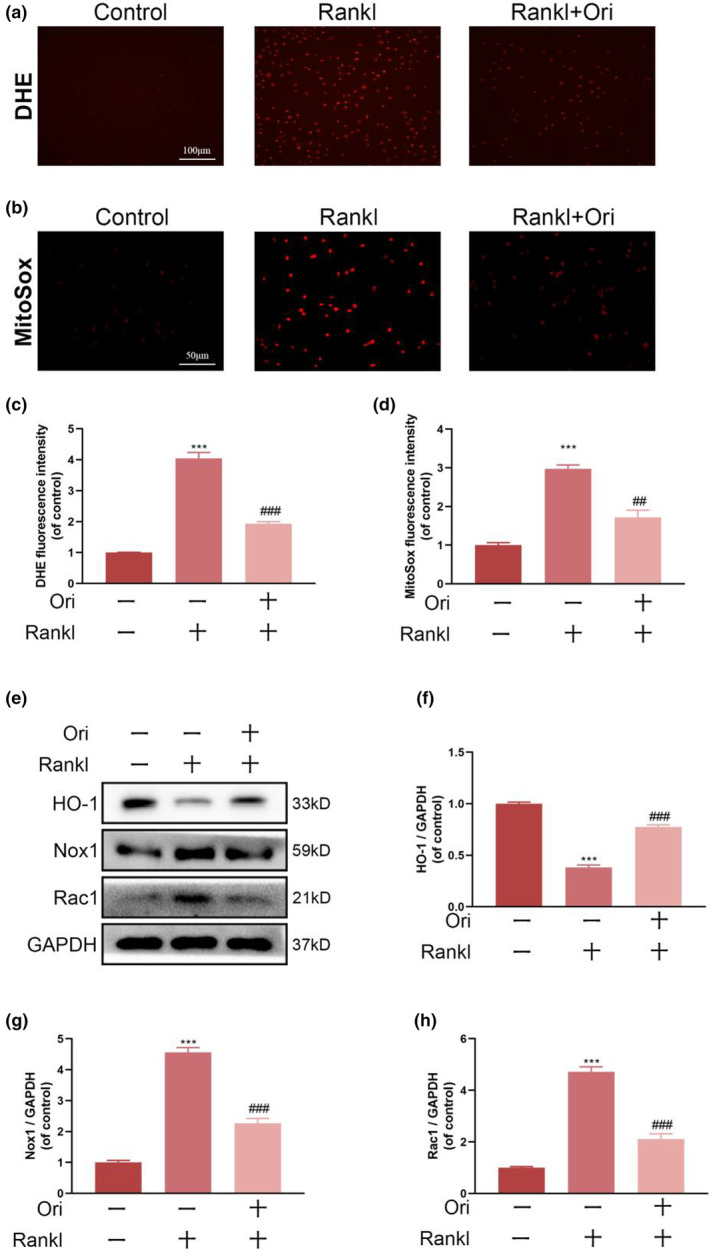
Orientin inhibits RANKL‐induced ROS production and stimulates the expression of antioxidation‐related proteins. (a and c) DHE fluorescence of differently treated BMMs, shown in representative photographs and analyzed quantitatively. (b and d) MitoSox fluorescence was photographed and measured quantitatively in differently treated BMMs. (e–h) In order to determine how HO‐1, Nox1, and Rac1 protein expression levels relate to antioxidation in the variously treated BMMs, western blotting was used. The numbers in the figures are the means ± SEM of three independent measurements. Statistical significance between the test and control groups: ***p < .001. When compared to the RANKL groups, ^##^ *p* < .01, ^###^ *p* < .001.

**FIGURE 4:**
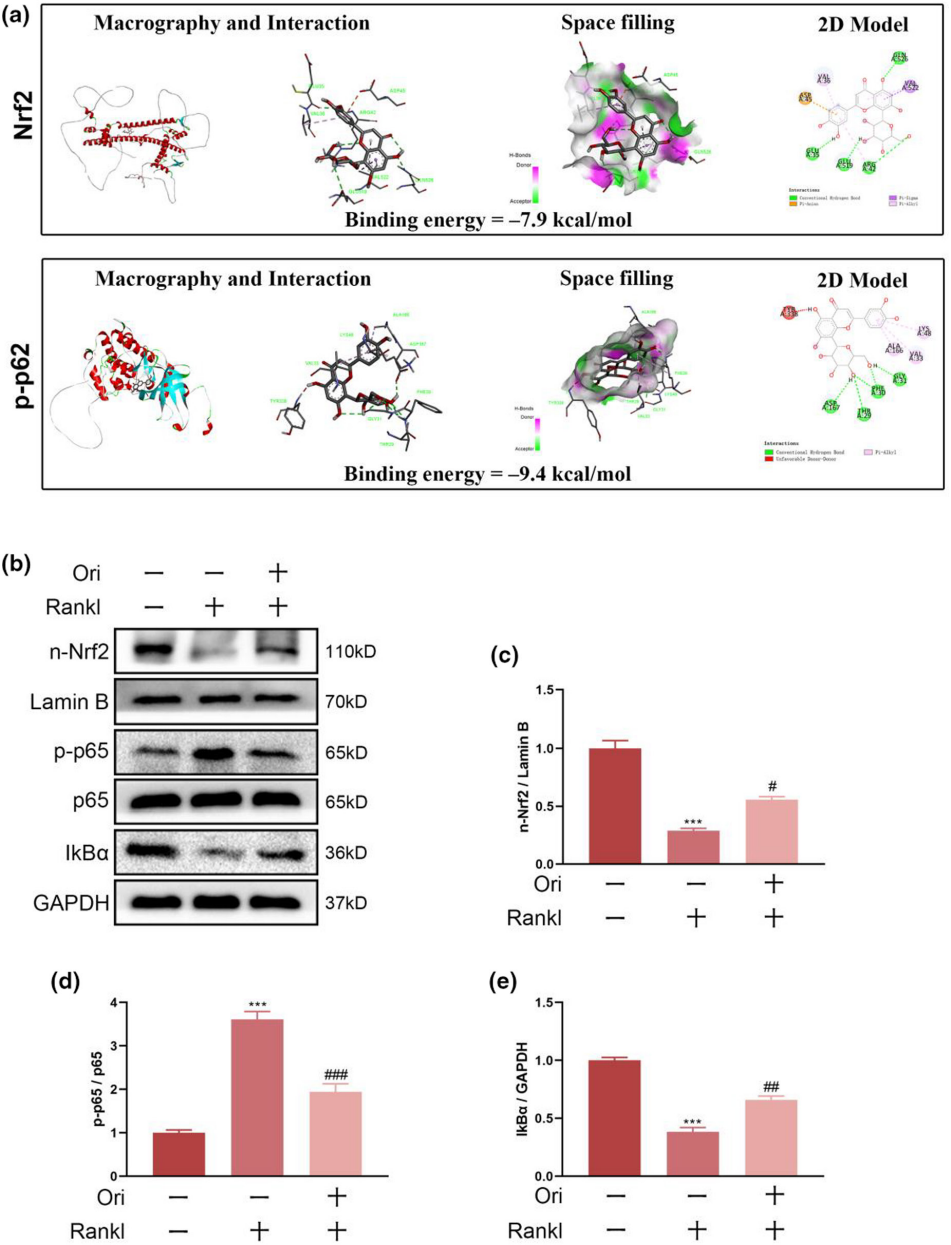
In vitro studies have shown that orientin inhibits osteoclastogenesis via activating the Nrf2/NF‐κB signaling pathway. (a) The protein residues in a 3D binding model are shown using a ribbon model. Docking Ori with either Nrf2 or p‐p65 reveals binding sites with affinities of 7.9 and 9.4 kcal/mol. Using a space‐filling model, we can see that Ori binds to the pockets for Nrf2 and p‐p65. (b–e) Western blotting examination of n‐Nrf2, p‐p65, and IKBa protein expression in the differently treated BMMs. The numbers in the figures are the means ± SEM of three independent measurements. Statistical significance between the test and control groups: ***p < .001. When compared to the RANKL groups, ^#^ *p* < .05, ^##^ *p* < .01, ^###^ *p* < .001.

ROS plays a crucial role in the formation of osteoclasts in response to RNAKL stimulation, as evidenced by a growing body of research. We, therefore, investigated whether Ori can influence ROS production in BMMs. A DHE dye was used to detect intracellular ROS production, and Nikon microscopes were used to record DHE fluorescence. The Ori treatment group showed a significant reduction in the intensity of DHE fluorescence per positive cell, as compared to the RANKL treatment group. (Figure 3a,b). In addition, MitoSox Red probes were adopted for examining the ROS levels in mitochondria in osteoclasts. Compared with the Rankl group, Ori significantly decreased MitoROS levels. Furthermore, HO‐1, Nox1, and Rac1 were oxidative stress‐related proteins. Nox1 and Rac1 expression was substantially increased in Rankl‐induced BMMs, but decreased in Ori + Rankl‐induced BMMs, as determined by western blotting. In contrast, HO‐1 expression was elevated in Rankl‐identified BMMs treated with Ori (Figure 4).

**FIGURE 5:**
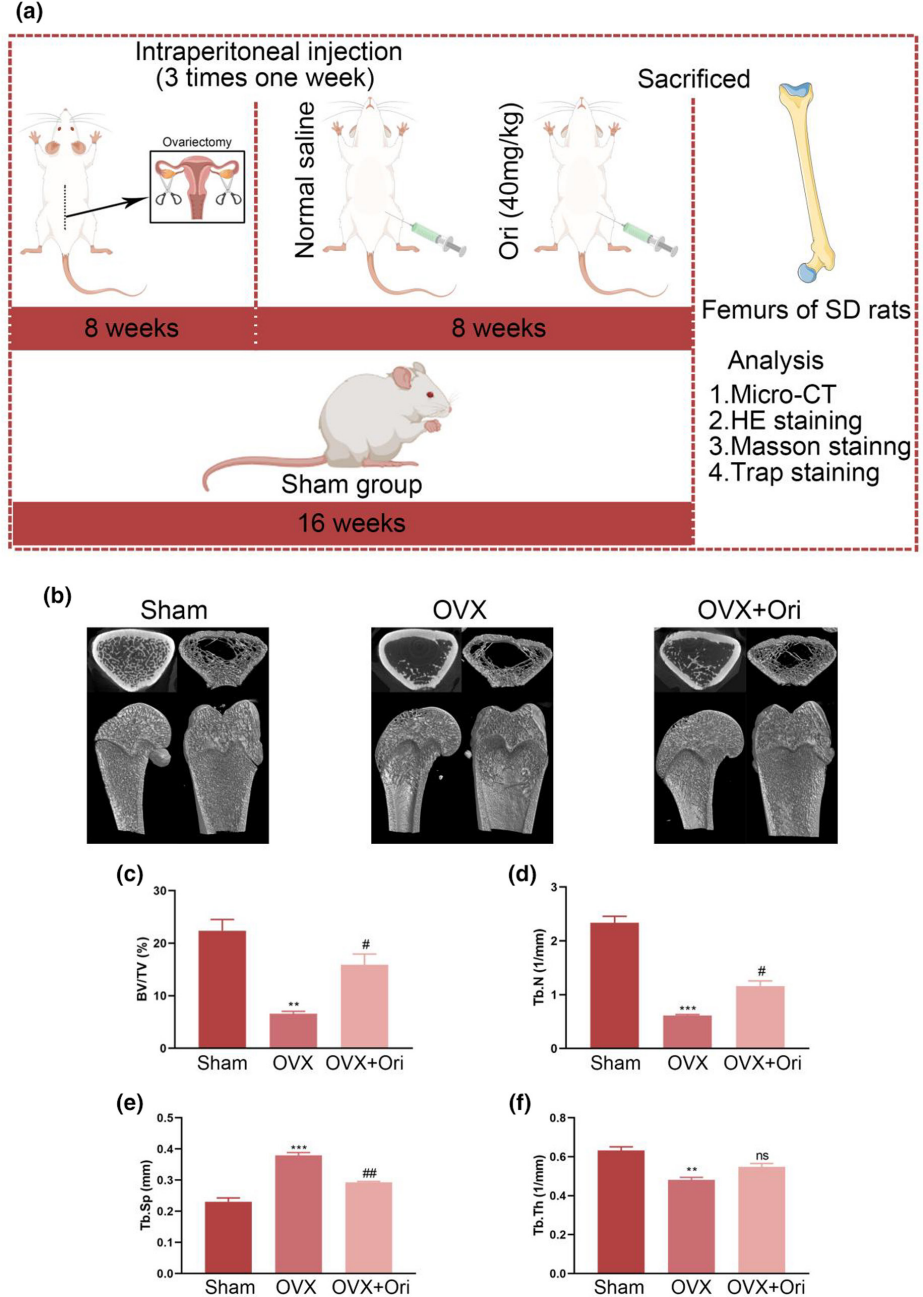
Orientin therapy reduces the bone loss caused by ovariectomy. (a) Rats undergoing therapy and administration. (b) Micro‐CT scanning was used to capture images of distal femur longitudinal and transverse slices. (c–f) Data from animals given various doses of a drug are shown for BV/TV, Tb. Sp, Tb. N, and Tb. Th. The numbers in the figures are the means ± SEM of three independent measurements. **p < .01, ***p < .001 vs. Sham groups. ^#^ *p* < .05, ^##^ *p* < .01 vs. OVX groups. ns = no significance.

Following this, we looked into how Ori influences NF‐κB/Nrf2 activation. We used molecular docking analysis to identify potential interactions between Ori and proteins in the NF‐κB/Nrf2 pathway. There are Ori binding sites in the Nrf2 and p‐p62 proteins (Figure 5a). Molecular docking experiments demonstrate that the molecule has a potent binding impact with target proteins, with binding energies of −7.9 and − 9.4 kcal/mol, respectively. This is because the lower the energy of the drug and the target, the stronger the binding. In addition, analysis of signaling pathway protein expression was performed by co‐stimulating BMMs with 50 ng/mL RANKL and 40 μM Ori for 24 h, Western blotting analysis showed that Nrf2 in nuclear and Iκ‐Ba levels were significantly lower in the Rankl‐induced BMMs, whereas Ori treatment increased the levels of these proteins' levels. Contrarily, the expression of p‐p65 was decreased with Ori treatment in Rankl‐induced BMMs. According to these findings, Ori prevents the development of osteoclasts by stimulating the Nrf2 and NF‐κB pathways.

### 3.4 Ori protects rats from ovariectomy‐induced bone loss

We created an ovariectomy‐induced osteoporosis rat model to investigate the potential therapeutic effects of Ori on the disease. The model and administration in OVX rats were as stated (Figure 5a). The reconstructed 2D and 3D pictures showed that OVX caused significant bone loss in the OVX rats and that treatment with Ori significantly reduced this loss (Figure 5b). Quantitative analysis showed that BV/TV and Tb. N was significantly higher in the Ori treatment group compared to the OVX treatment group, whereas Tb. Sp was lower in the Ori treatment group. However, it is important to note that no statistically significant difference in trabecular thickness (Tb. Th) was observed between the OVX and OVX + Ori groups, as depicted in Figure 5f.

### 3.5 Ori treatment improves bone microarchitecture and promotes bone synthesis in vivo

**FIGURE 6:**
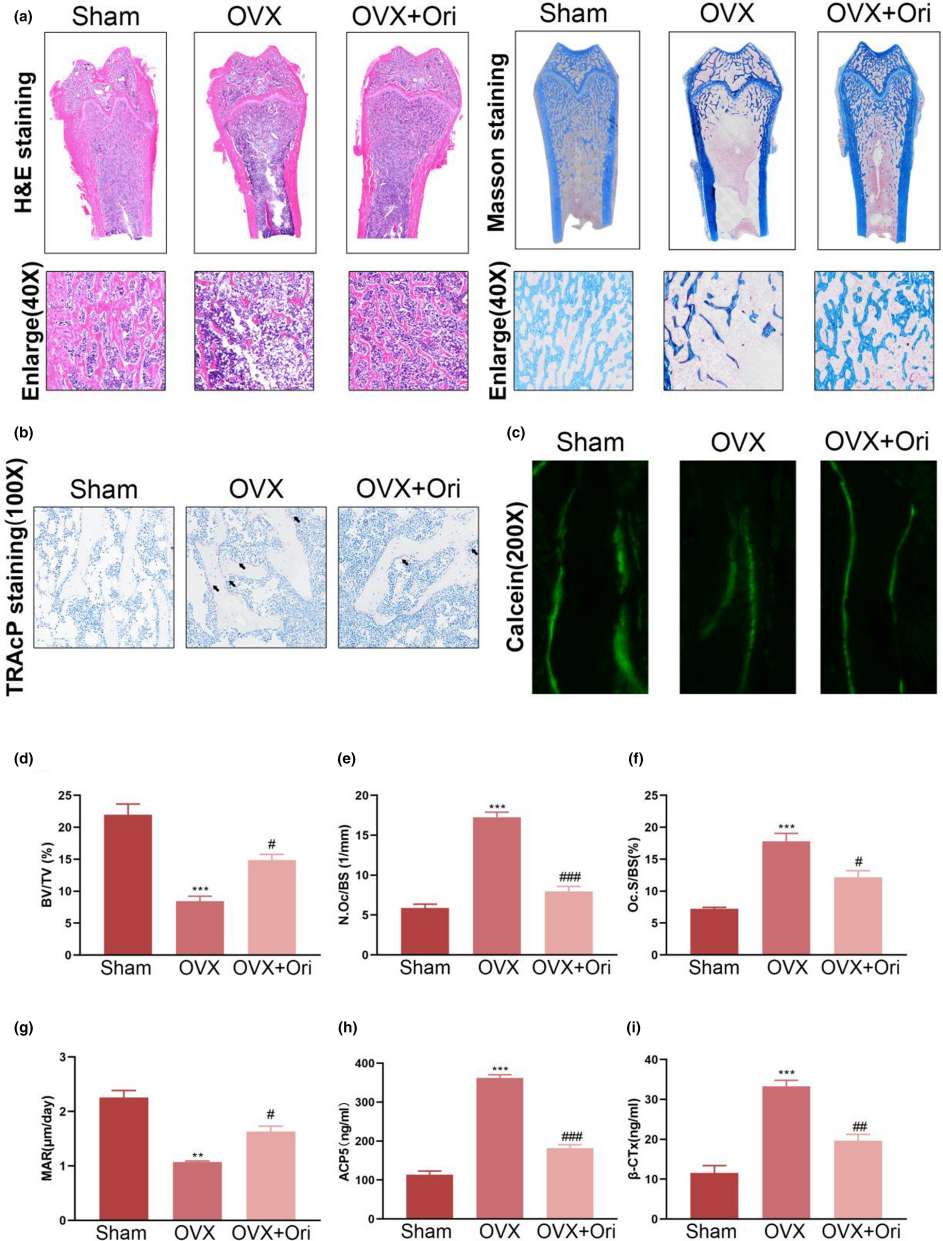
The microstructure was restored, and bone production was enhanced, in the treated rats. (a and d) Sample photos and statistical breakdown of H&E and Masson staining for various groups. (b, e, and f) Scale bar = 400 m. Distal femurs stained with TRAcP and analyzed quantitatively for Oc. S/BS and N. Oc/BS (scale bar: 400 μm). (c and g) The quantitative examination of the MAR and successive fluorescence labeling of calcein confirmed the presence of new bone growth. (h,i) Serum ACP5 and β‐CTx concentrations were measured quantitatively in mice. The numbers in the figures are the means ± SEM of three independent measurements. **p < .01, ***p < .001 vs. Sham groups. ^#^ *p* < .05, ^##^ *p* < .01, ^###^ *p* < .001 vs. OVX groups.

Furthermore, the suppressive impact of Ori on osteoclast formation was validated using H&E, Masson, and TRAcP staining, and Calcein fluorescence was used to determine Ori's impact in stimulating bone formation in vivo (Figure 6a,b). When comparing the OVX group to the OVX + Ori group, the findings indicated that the OVX + Ori group had a much higher BV/TV value (Figure 6d). Both N. Oc/BS and Oc. S/BS were elevated in OVX mice, as is seen in Figure 6e,f, and their levels were significantly lowered by further Ori. Bone morphological changes were also consistently reflected by the double‐labeling of calcein, as expected (Figure 6c,g). Moreover, Ori therapy reduced the hypersecretion of factors (ACP5 and β‐CTx) associated with osteoclasts that had been induced by OVX (Figure 6h,i). In conclusion, Ori can counteract the effects of an inadequate estrogen supply on bone health.

## 4 DISCUSSION

The global community has identified osteoporosis as a major health issue. It is estimated that over 200 million individuals globally are currently living with osteoporosis (Li et al., 2019). That figure is steadily growing as a result of the dramatic increase in the elderly population and the trend toward higher life expectancy (Yi et al., 2021). Postmenopausal women are at a higher risk for developing osteoporosis due to abnormal bone remodeling brought on by a lack of estrogen and an increase in ROS in vivo, both of which have a profoundly suppressive effect on osteoclastogenesis and osteoclast activity. The etiology of the illness is heavily dependent on the abnormal activation of osteoclasts. In order to effectively treat osteolytic disorders, it is crucial to target osteoclast activity and suppress ROS formation (Zhang, Cui, et al., 2020). Estrogen and bisphosphonate medications are now available for the treatment of osteoporosis, although they both come with potentially adverse effects. That is why it is important to keep looking for a new treatment for osteoporosis.

Natural flavonoid compounds are increasingly being recognized for their ability to reduce osteoclast activity and ameliorate critical osteoclastogenesis pathways (Kim et al., 2020). Fenugreek's major bioactive component, Orientin, is a C‐glycosyl flavonoid with numerous bioactivities and therapeutic benefits, including anti‐inflammatory, antidiabetes, antioxidative, and autophagy‐inducing properties (Kim et al., 2020; Thangaraj et al., 2019; Zhang et al., 2022). Zhang confirmed that in vitro, Ori could inhibit TBHP‐induced oxidative stress in cells from the rat nucleus pulposus (Zhang et al., 2022). In addition, Li and Kong et al. reported that it possesses antioxidation, antiapoptosis, and anti‐inflammatory properties (Kong et al., 2020; Li et al., 2022). Considering its crucial role in several metabolic diseases, Ori may directly participate in bone metabolism, especially in osteoclastogenesis. Our data suggested that Ori has the ability to suppress osteoclast development in vitro and in vivo, hence reducing bone loss in OVX rats. Possible mechanisms of antioxidants of Ori were also described. Firstly, TRAcP staining and bone resorption pit assays revealed the inhibitory impact of Ori at 40 μM on osteoclastogenesis in vitro. It is widely known that in the presence of RANKL, mononuclear osteoclast precursors merge and develop into bone‐resorbing multinuclear osteoclasts. Thus, osteoclast fusion crucially impacts the bone resorption mediated by osteoclast. A large F‐actin ring is formed on the fused multinuclear cells in the process of osteoclastogenesis, enabling bone resorption. In our study, Ori could lower the F‐actin ring size and quality, proving that Ori restrained osteoclast function in vitro. In addition, animal experiments also served to verify the treatment effect of Ori in vivo, which improved the bone loss of OVX rats in vivo.

Translocation of Nrf2 into the nucleus, where it eventually activates downstream antioxidant enzymes to lower ROS levels, has been linked to its role in regulating osteoclast development and bone resorption function (Yang et al., 2020). The net amount of intracellular ROS is determined by the ratio of ROS production to the amount of ROS cleared by the intracellular antioxidant system, which is mediated by Nrf2 (Escaffre et al., 2015). It has been observed that antioxidants may prevent osteoclast development by decreasing ROS levels and increasing Nrf2/HO‐1 pathway activity (Zhang, Shang, et al., 2020). While Nrf2 activation decreases antioxidant enzyme expression and reduces intracellular ROS levels in osteoclasts, inactivation of Nrf2 increases intracellular ROS levels and promotes osteoclastogenesis (Yoon et al., 2016). To reduce H_2_O_2_‐induced oxidative damage in RAW264.7 cells, Ori was shown to activate the antioxidant‐related proteins GCLM, GCLC, HO‐1, and NQO1, in addition to clearly enhancing Nrf2 nuclear translocation, according to Xiao et al (Xiao et al., 2021); Lawal et al. found that Ori modulates the oxidative stress associated injury of diesel exhaust particles in human umbilical vein endothelial cells (Lawal et al., 2019). Our data demonstrated that Ori inhibited osteoclastogenesis by activating the Nrf2‐mediated intracellular antioxidant system (HO‐1, Nox1, and Rac1). One of the most important signaling pathways involved in osteoclastogenesis is NF‐κB signaling. Transcription of target genes like cyclin‐dependent kinase inhibitor (CTSK) and cyclin‐dependent kinase inhibitor (c‐Fos) is stimulated by the phosphorylation of NF‐κB signaling proteins. In BMMs, we found that treatment with Ori suppressed IB degradation and p65 phosphorylation. These results suggest that the underlying molecular mechanism via which Ori suppresses osteoclastogenesis may be identified.

Unfortunately, this research has a few caveats. First, the causes and progression of osteoporosis are complex and include a variety of factors. However, in our investigation, we only looked at the NF‐κB/Nrf2 axis. Second, whether Ori acts directly on ROS through the NF‐κB/Nrf2 pathway or if there are certain molecular targets connected to mitochondrial ROS and NF‐κB/Nrf2 is unknown. Furthermore, gene knockdown and silencing, which are more convincing, were not used in the research. Overall, our work suggests a course for future osteoporosis research and therapy.

## 5 CONCLUSION

**FIGURE 7:**
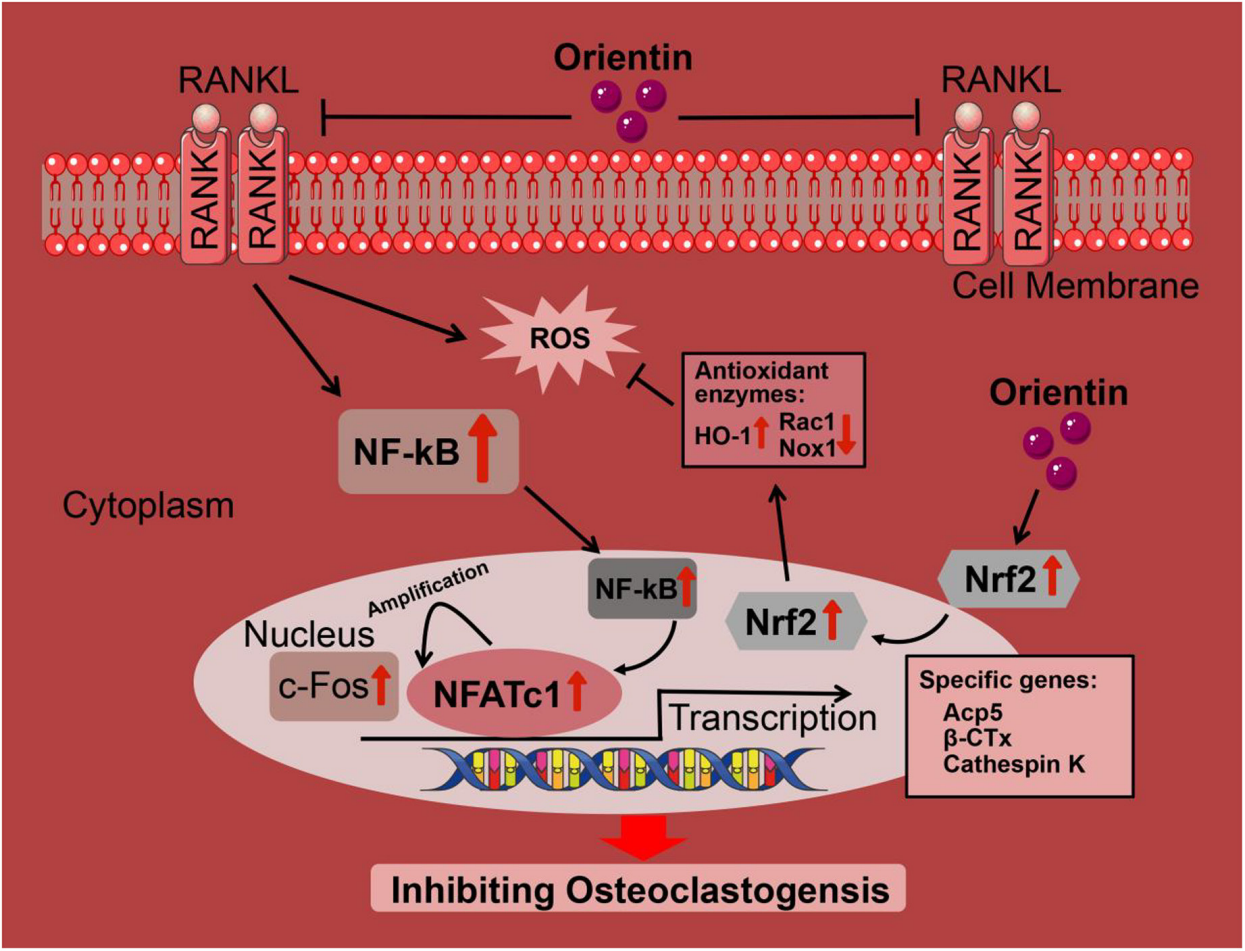
Illustration of Ori's modulatory role in RANKL‐induced intracellular signaling cascades.

To begin, we showed that Ori, a flavonoid component derived from natural plants, suppresses ROS generation and attenuates OVX‐induced osteoporosis in vivo by inhibiting osteoclastogenesis in vitro. Specifically, Ori suppressed NF‐κB signaling pathways and increased the expression of Nrf2 and other antioxidant genes (Figure 7). The OVX rat model also demonstrates an antiosteolytic property of Ori mediation. To sum up, our results suggest that, from a cellular and molecular standpoint, Ori is a promising therapeutic candidate for treating bone loss illnesses such as osteoporosis and osteolysis by inhibiting osteoclast development induced by RANKL.

## AUTHOR CONTRIBUTIONS

**Yan Zheng:** Data curation (equal); resources (equal); writing – original draft (lead); writing – review and editing (lead). **Xing Wang:** Investigation (equal); supervision (equal); visualization (equal). **Ya‐Jing Pan:** Formal analysis (equal); methodology (equal); project administration (equal). **Xiao‐Feng Shi:** Data curation (equal); software (equal). **Lei Yang:** Funding acquisition (lead). **Yong‐Liang Lou:** Conceptualization (lead).

## FUNDING INFORMATION

This work was supported by the National Natural Science Foundation of China (No. 82072310) and Traditional Chinese Medicine of Zhejiang Province Science and Technology plan project (No. 2023010569).

## CONFLICT OF INTEREST STATEMENT

The authors declare that they have no known competing financial interests or personal relationships that could have appeared to influence the work reported in this paper.

## Data Availability

Data will be made available on request.

## APPENDIX A

### A.1

The genes' primer sequences were as follows: NFATc1‐forward: 5′‐CAACGCCCTGACCACCGATAG‐3′; NFATc1‐reverse: 5′‐GGCTGCCTTCCGTCTCATAGT‐3′; c‐Fos‐forward: 5′‐GCGAGCAACTGAGAAGAC‐3′, c‐Fos‐reverse: 5′‐TTGAAACCCGAGAACATC‐3′; CTSK‐forward: 5′‐GGGAGAAAAACCTGAAGC‐3′, CTSK‐reverse: 5′‐ATTCTGGGGACTCAGAGC‐3′; and GAPDH‐forward: 5′‐GGTGAAGGTCGGTGTGAACG‐3′, GAPDH‐reverse: 5′‐CTCGCTCCTGGAAGATG GTG‐3′.
